# A Rare Encounter With Peripheral Dentinoma: Clinicopathological Insights

**DOI:** 10.7759/cureus.116258

**Published:** 2026-09-15

**Authors:** Baji Libujaa, N. P. Rajiv, Priya N Sanjeevarayappa

**Affiliations:** 1 Department of Periodontics, Vokkaligara Sangha Dental College & Hospital, Bangalore, IND; 2 Department of Oral and Maxillofacial Pathology, Vokkaligara Sangha Dental College & Hospital, Bangalore, IND

**Keywords:** ameloblastic fibrodentinoma, dentinoma, mixed odontogenic tumor, odontogenic tumor, peripheral dentinoma

## Abstract

Odontogenic tumors comprise a diverse group of lesions arising from tooth-forming tissues and often show overlapping clinical, radiographic, and microscopic features, creating diagnostic challenges. Peripheral dentinoma is an exceptionally rare dentin/dentinoid producing odontogenic tumor characterized by dysplastic dentin or dentinoid formation, with odontogenic epithelial and ectomesenchymal components that may vary according to the histologic pattern. We report a case of peripheral dentinoma in a 24-year-old male patient in the lower right posterior tooth region. Radiographic examination revealed a well-defined radiopaque lesion in relation to the mesial aspect of 46, and a provisional diagnosis of a foreign body was considered. The lesion was surgically excised and submitted for histopathological evaluation. Microscopy showed a disorganized mass of tubular dentin along with sclerotic, atubular eosinophilic dentinoid areas, suggestive of dentinoma. This case highlights the rarity of peripheral dentinoma and the importance of clinicopathological correlation for accurate diagnosis, appropriate management, and follow-up.

## Introduction

Dentinoma is a rare odontogenic tumor characterized by the presence of dysplastic dentin in association with immature connective tissue and odontogenic epithelium [1]. Historically, the terms ameloblastic fibrodentinoma (AFD) and fibroameloblastic dentinoma have also been used in relation to dentinoma [2]. No specific sex predilection has been observed, and most reported cases have occurred in the mandible of young adults [1]. The first case was published in 1936 by F. E. Straith [3]. According to the 1958 classification of odontogenic tumors proposed by Pindborg and Clausen, dentinoma was considered an epithelial tumor with inductive potential [4]. Based on the presence or absence of odontogenic epithelium, along with variations in the microscopic appearance of the stromal component and hard tissue, dentinoma has been further classified into immature and mature subtypes [5,6].

The terminology of dentin-producing odontogenic lesions has evolved with successive classifications. Histologically, ameloblastic fibrodentinoma is characterized by ameloblastic epithelium associated with dental-papilla-like areas and dysplastic dentin, whereas ameloblastic fibro-odontoma contains ameloblastic fibroma-like areas along with varying proportions of dentin and enamel. Odontomas, in contrast, demonstrate odontogenic epithelium with organised/ disorganised tooth like structures [7]. Although ameloblastic fibrodentinoma and ameloblastic fibro-odontoma were included within the mixed odontogenic tumors in the 2005 World Health Organisation (WHO) classification, they were subsequently removed as separate entities in the 2017 classification, an approach retained in the 2022 WHO classification, because most such lesions are considered to fall within the spectrum of developing odontomas; however, the possibility that some represent true neoplasms remains debated [8].

Peripheral presentation of a dentinoma is exceptionally uncommon. In the present case, the term “peripheral dentinoma” was used because the lesion presented as a localized extraosseous hard-tissue mass adjacent to the mandibular first molar, without evidence of a primary intraosseous component, while histopathological examination demonstrated characteristic dentin and dentinoid formation. The present case is therefore reported to highlight the unusual peripheral presentation of dentinoma, its potential to mimic more common localized gingival hard-tissue lesions, and the essential role of histopathological examination in establishing the definitive diagnosis.

## Case presentation

A 24-year-old systemically healthy male patient presented with the chief complaint of a decayed tooth in the lower right posterior region. He was referred to the Department of Periodontics for evaluation of the periodontal condition in relation to teeth 45 and 46. The patient had no pain or discomfort and was unaware of the lesion.

Intraoral examination revealed a small, hard, bone-like mass on the mesial aspect of tooth 46, partly situated within the gingival sulcus and partly projecting through the gingiva (Figure 1).

**Figure 1:**
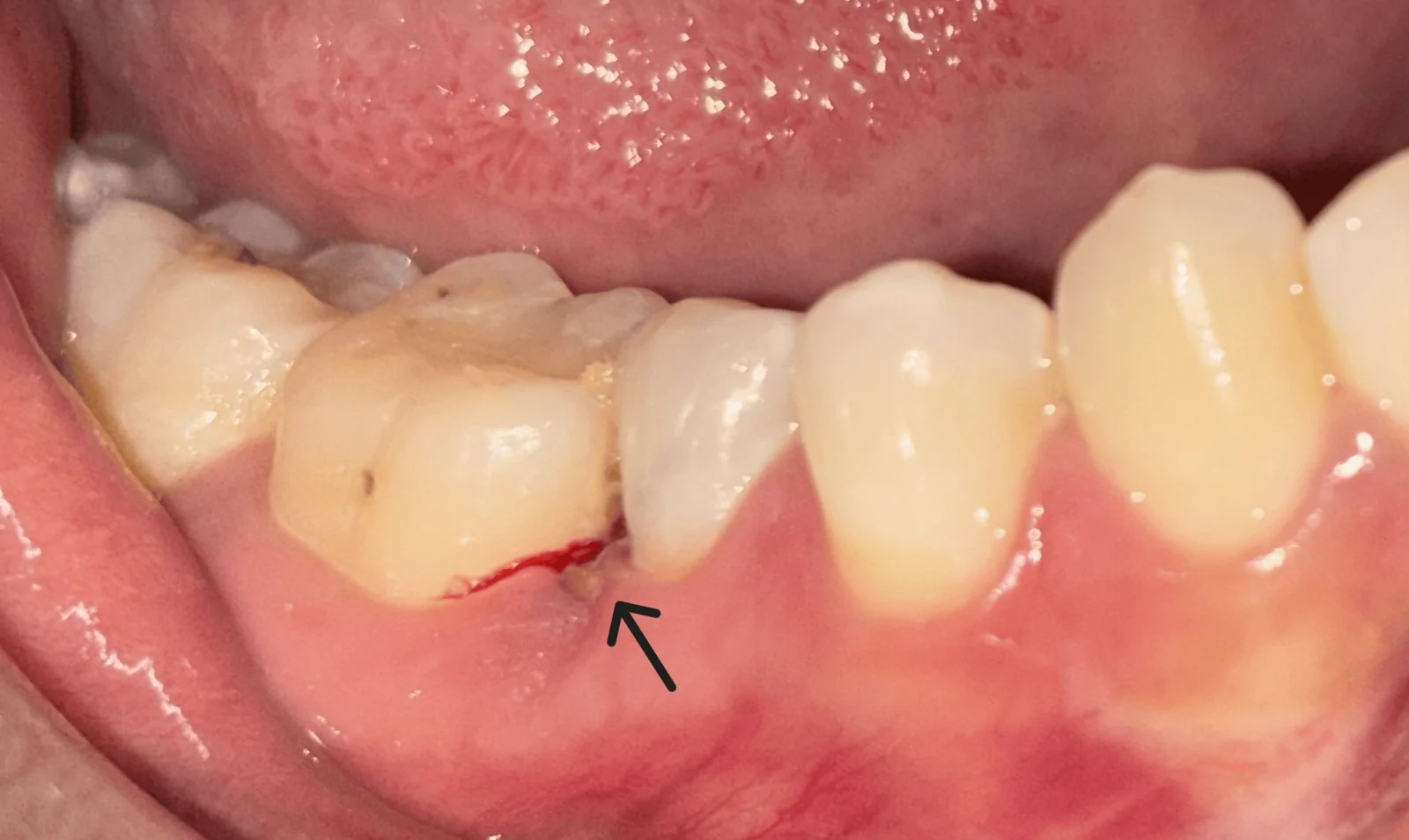
Preoperative clinical photograph showing the small, hard, bone-like mass on the mesial aspect of tooth 46, partly projecting through the gingival sulcus. The arrow indicates the lesion.

Mild gingival inflammation was present. Periodontal examination revealed a probing depth of 6 mm on the mesial aspect of 46, with bleeding on probing. No tooth mobility was noted in teeth 45 and 46, and both teeth were vital. An intraoral periapical radiograph demonstrated a well-defined radiopaque lesion mesial to 46, associated with an angular bone defect between teeth 45 and 46 (Figure 2).

**Figure 2:**
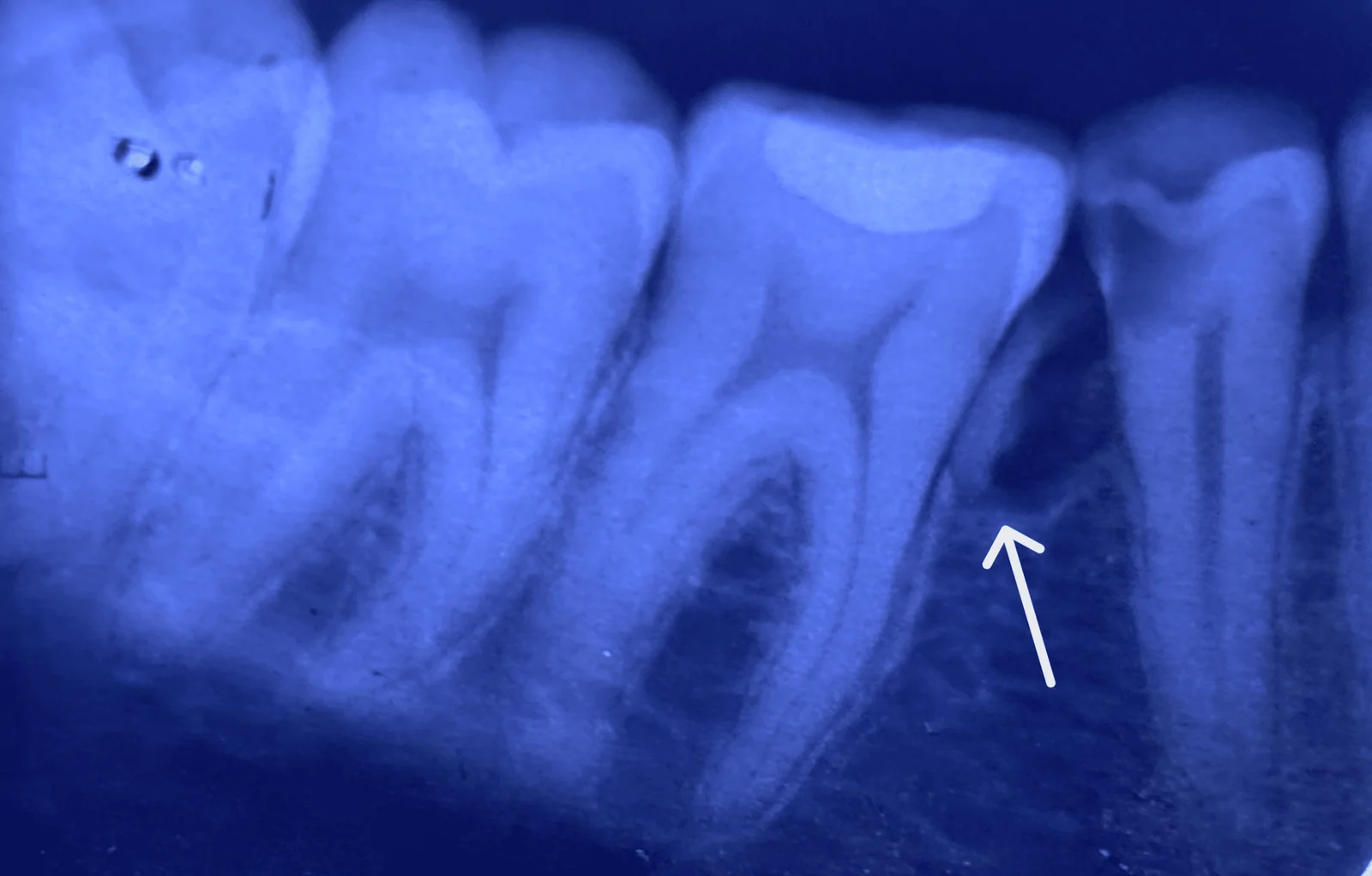
Intraoral periapical radiograph showing a well-defined radiopaque lesion mesial to tooth 46, with an angular bone defect between teeth 45 and 46. The arrow indicates the radiopaque lesion.

Initial periodontal therapy, including full-mouth scaling and root planing, was performed. Removal of the calcified mass using a curette was attempted, but the lesion was hard and firmly adherent to 46 within the sulcular area. Open flap debridement with surgical excision was planned.

After administration of local anesthesia with 2% lidocaine and 1:80,000 adrenaline, sulcular incisions were placed using a No. 15 blade, and buccal and lingual full-thickness flaps were reflected. The hard tissue mass was carefully removed with curettes, followed by thorough debridement and irrigation. The flaps were repositioned and sutured with simple interrupted sutures. A postoperative intraoral periapical radiograph confirmed complete removal. Periodontal dressing and postoperative instructions were given. Sutures were removed after one week; healing was uneventful, and follow-up at one and three months showed satisfactory healing.

The excised hard tissue mass was sent for histopathological evaluation. On gross examination, the excised hard tissue was round in shape and measured 0.3 cm x 0.2 cm. Microscopic examination of decalcified hematoxylin & eosin (H&E) stained sections of the hard tissue fragment showed a predominantly disorganized mass of tubular dentin with sclerotic, atubular eosinophilic dentinoid areas. A central area of fibrous tissue resembling pulp was also evident (Figures 3, 4). 

**Figure 3:**
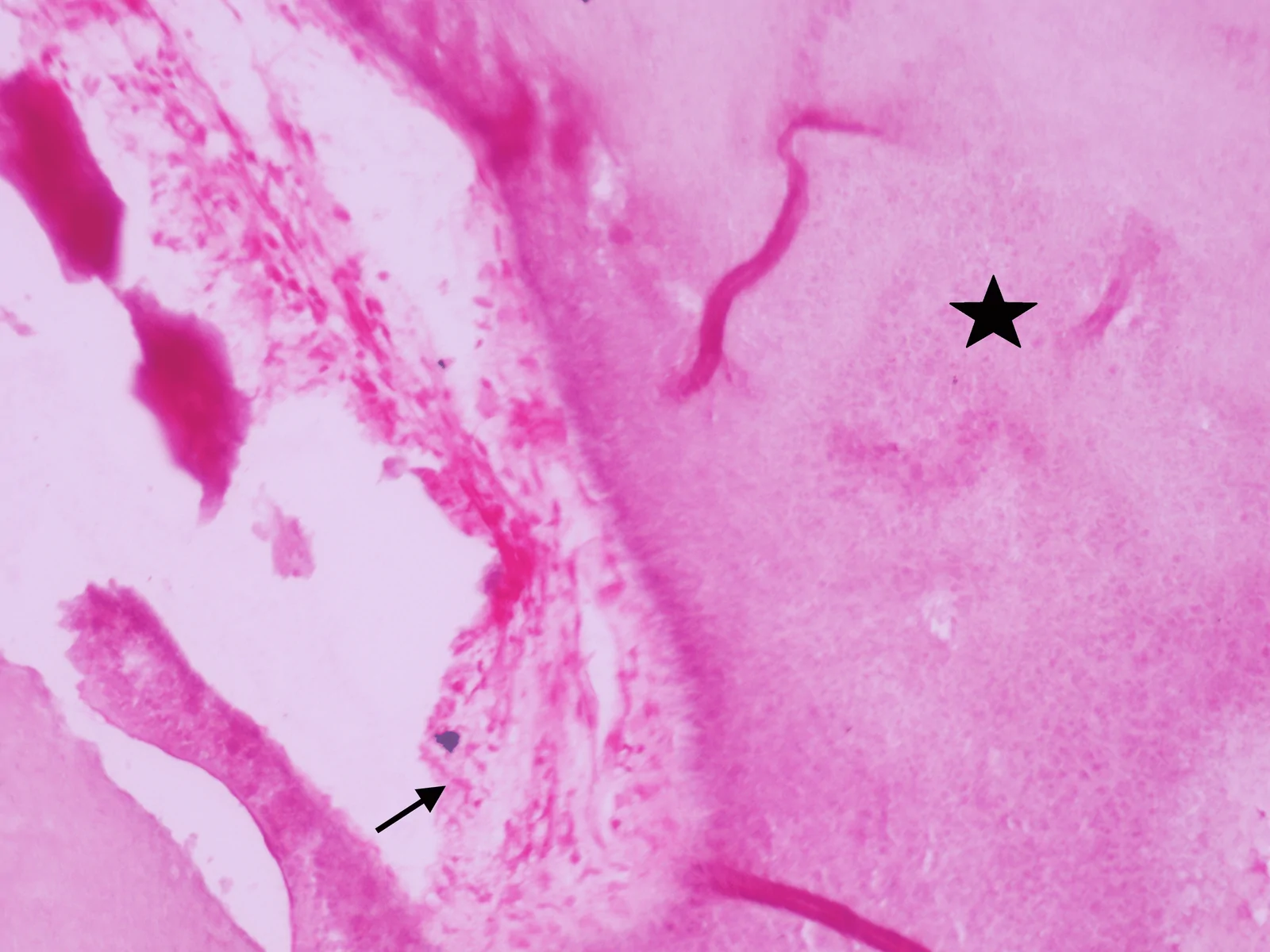
Photomicrograph showing disorganized dentin/dentinoid hard tissue (star) and adjacent pulp-like tissue (arrow). Hematoxylin and eosin stain, original magnification ×10.

**Figure 4:**
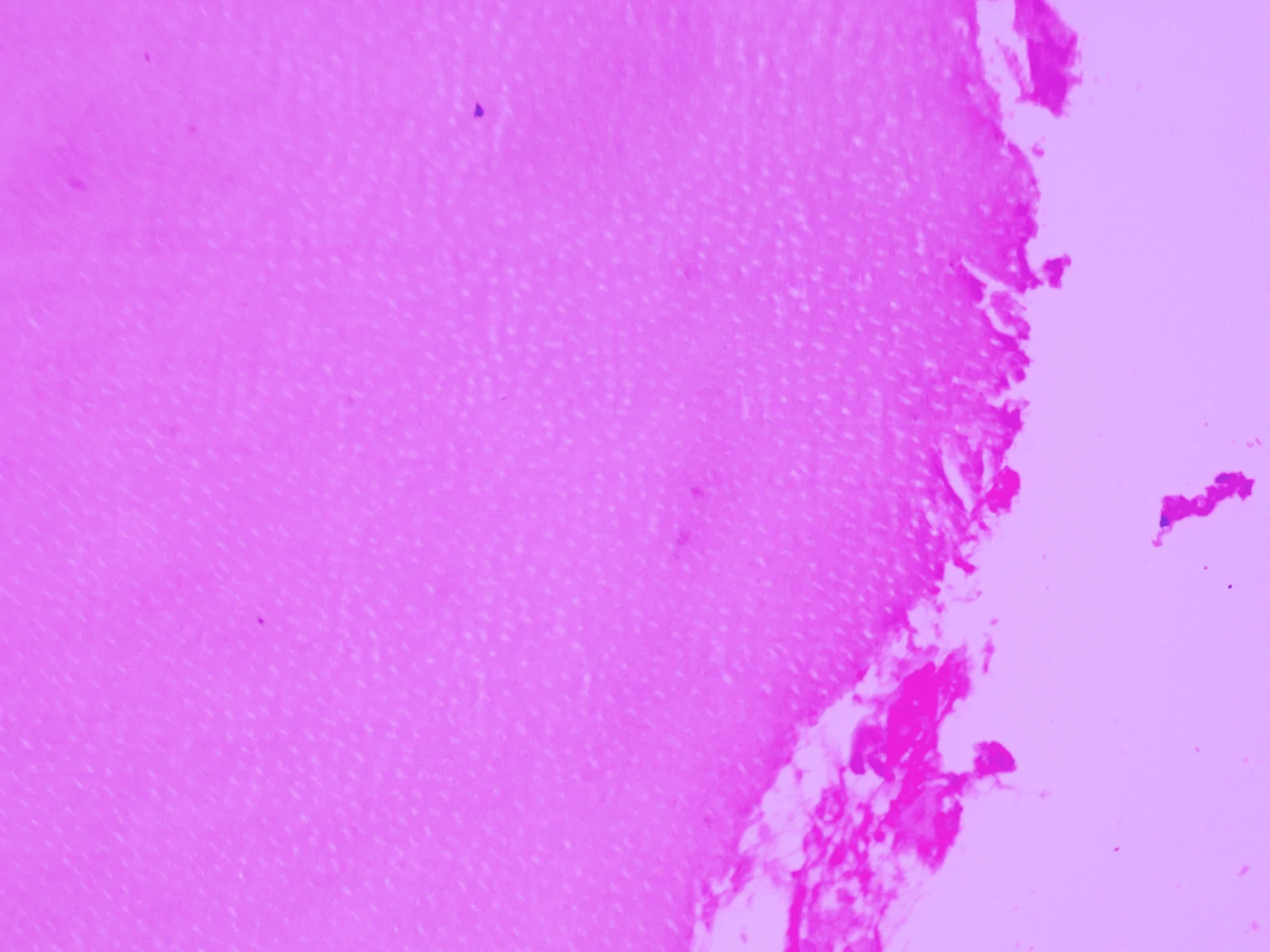
Photomicrograph showing tubular dentin with numerous identifiable dentinal tubules. Hematoxylin and eosin stain, original magnification ×20

Microscopic examination of the H&E-stained soft tissue sections revealed parakeratinized stratified squamous epithelium overlying moderately fibrous connective tissue. The connective tissue showed thin-walled endothelial-lined capillaries, focal areas of granulation tissue with lymphocytic aggregates, bacterial colonies, scattered dystrophic calcifications, fibrosis, hyalinization, and areas of hemorrhage.

The predominance of disorganized tubular dentin and dentinoid, together with the central pulp-like component, favored the diagnosis of dentinoma. Histologically, this pattern is distinct from odontoma, which demonstrates dental hard tissues arranged as organized tooth-like structures or as a disorganized odontogenic hard-tissue mass, and from historically described ameloblastic fibrodentinoma, which is characterized by ameloblastic odontogenic epithelium associated with primitive dental-papilla-like ectomesenchyme and dysplastic dentin [7]. In conjunction with the peripheral gingival location and the absence of a primary intraosseous lesion, the clinicopathological findings supported the diagnosis of peripheral dentinoma.

## Discussion

Dentinoma is a rare odontogenic lesion characterized by dentin, dysplastic dentin, or dentinoid-like hard tissue associated with odontogenic epithelial and ectomesenchymal components. It is among the rarest odontogenic tumors, with few reported cases [9]. Anker and Radden described it as a neoplasm composed of dysplastic dentin, immature connective tissue and odontogenic epithelium, with no definite sex predilection and greater mandibular predilection in young adults [1].

The classification and histogenesis of dentinoma remain controversial. Under the 2022 WHO classification, dentinoma does not appear as an independently recognized odontogenic tumor [8], and the term is now mainly used in a historical or descriptive context for lesions exhibiting dentin or dentinoid formation. Historically, considerable variation has existed in the terminology and classification of dentinoma and ameloblastic fibrodentinoma [10]. In the 2017 WHO classification, ameloblastic fibrodentinoma and ameloblastic fibro-odontoma were no longer recognized as separate entities, as many such lesions were considered to represent developing odontomas. This approach has been retained in the 2022 WHO classification; however, the possibility that at least some of these lesions represent true neoplasms remains debated [8]. Pindborg and Clausen classified dentinoma as an epithelial odontogenic tumor with inductive activity, while later authors emphasized epithelial-mesenchymal interaction in dentin-like tissue formation [4]. Lukinmaa et al. suggested that dentinoid may form by hyalinization and mineralization of collagen following odontogenic epithelial induction, while epithelial degeneration may explain its absence in some cases [5]. The present findings support this concept.

Clinically, dentinomas are usually reported in young individuals and commonly involve the posterior mandible [1,5]. They may be incidental or associated with swelling, pain, impacted teeth, or local inflammation [8]. Peripheral presentation is particularly uncommon; in a systematic review of 64 ameloblastic fibrodentinomas, only four cases occurred in a peripheral location [11]. The current case occurred in a 24-year-old male patient in the mandibular posterior region, consistent with reported age and site. However, its small, hard, bone-like mass partly within the gingival sulcus and projecting through the gingiva was unusual and mimicked a foreign body or calcified gingival mass. A peripheral gingival presentation has also been described by Minamizato et al., who reported a peripheral-type ameloblastic fibrodentinoma with features of so-called “immature dentinoma,” highlighting the clinicopathological variability of these rare peripheral lesions [12].

Radiographic appearance depends on hard tissue amount and pattern. Reported cases include radiopaque, radiolucent, or mixed lesions, sometimes with impacted teeth. Hoggins and Browne described a demarcated radiopaque mandibular mass [9], Anker and Radden a mixed lesion [1], and Lukinmaa et al. a multilocular radiolucency with irregular radiopaque areas [5]. In the current case, the intraoral periapical radiograph showed a well-defined radiopaque lesion mesial to 46 with an angular defect between 45 and 46, supporting surgical exploration and histopathological examination.

Histopathology is essential because dentinoma may contain tubular dentin, osteodentin, dentinoid, hyalinized connective tissue, and odontogenic epithelial islands or strands [1,13]. Similar features were reported by Hoggins and Browne [9] and Lukinmaa et al. [5]. In the current case, disorganized tubular dentin, sclerotic atubular dentinoid areas, and central pulp-like fibrous tissue supported the diagnosis of dentinoma.

The main differentials include ameloblastic fibrodentinoma, ameloblastic fibro-odontoma, and developing odontoma. Ameloblastic fibroma lacks appreciable hard tissue, while enamel matrix favors ameloblastic fibro-odontoma or odontoma [1]. Diagnostic difficulty persists because dentinoma overlaps with other dentinoid-containing lesions [9]. In the present case, the absence of enamel and the presence of tubular dentin and dentinoid favored dentinoma.

Dentinoma has been described as mature or immature based on odontogenic epithelium, dentin formation, odontoblast-like cells, and stromal maturation, although this is not uniformly accepted [4,13]. Exact subtyping was difficult, but following correlation of the histopathological features with the clinical and radiographic findings, a diagnosis of peripheral dentinoma was favored.

Conservative surgical removal is the treatment of choice. Uneventful healing and absence of recurrence have been reported after excision or enucleation [5,9]. In this case, open flap debridement with excision resulted in satisfactory healing and no symptoms at the three-month follow-up, emphasizing histopathological evaluation of unusual gingival or sulcular hard tissue masses.

## Conclusions

Peripheral dentinoma is an exceptionally rare odontogenic lesion that may present with nonspecific clinical and radiographic features, making diagnosis challenging. In the present case, the lesion clinically resembled a foreign body or localized calcified mass in the gingival sulcus, while histopathological examination demonstrated characteristic tubular dentin and dentinoid formation. Histopathologically, it should be distinguished from other dentin-producing odontogenic lesions, particularly odontoma and historically related entities such as ameloblastic fibrodentinoma. This case emphasizes the importance of histopathological evaluation of unusual hard-tissue masses in the periodontal region and the role of clinicopathological correlation in establishing an accurate diagnosis.
